# Advances in water intake assessment

**DOI:** 10.1007/s00394-015-0957-3

**Published:** 2015-06-16

**Authors:** Jodi Dunmeyer Stookey, Juergen Koenig

**Affiliations:** Children’s Hospital Oakland Research Institute, 5700 Martin Luther King Jr. Way, Oakland, CA 94609 USA; Department of Nutritional Sciences, University of Vienna, Vienna, Austria

 In the 1990s, water intake was not a priority area for nutrition research. Water intake data were used by public health dentists to inform fluoridation initiatives, and by cancer and infectious-disease epidemiologists to estimate the risk of water-borne contaminants and carcinogens. Nutrition researchers, in reaction to the global obesity epidemic, focused data collection on macronutrient and energy intake, without tracking water.

Over the past decade, nutritionists’ interest in water intake has grown along with increasing awareness of the effects of beverage consumption on obesity and chronic disease risk. The World Health Organization, European Food Safety Authority (EFSA), the American Medical Association, US Centers for Disease Control, and national Dietary Guidelines in many countries began recommending drinking water instead of caloric beverages to reduce obesity and chronic disease risk. Debate over water intake recommendations drew attention to gaps in the water intake evidence base.

The articles in this supplement describe recent methodological and substantive developments in nutrition research on water intake and outline remaining gaps in knowledge.

Gandy [1] describes inconsistent water intake assessment between countries, a lack of validated methods, and the need to standardize international efforts to estimate water intake. Water intake assessment methods have yet to be validated against objective biomarkers, such as water turnover. Unknown potential for measurement error limits interpretation of existing water intake data.

Bardosono et al. [2] explore the potential for error in water intake estimates derived by 24-h recall. Although one 24-h diet recall is generally understood to adequately estimate the energy and macronutrient intake of a group, these authors report significant underestimation of *absolute* beverage intake for groups of adolescents and adults, relative to a 7-day diet record. One 24-h recall appears to only yield valid estimates of *relative* fluid intake for groups, i.e. drinking water expressed as a percentage of total fluid intake. Although the data support use of one 24-h recall to evaluate interventions promoting relative beverage change, i.e. drinking water *instead of* caloric beverages, at the community- or group-level, it remains to be determined if and how sources of error in water intake estimation can be reduced.

While a 7-day record appears to be a more reliable method to capture fluid intake, the burden associated with this instrument may impact its usability and acceptance in the general population. Monnerie et al. [3] provide data of a study comparing an online version of a 7-day record with a paper version of this instrument. They report that the online version resulted in higher water intake from fluids and a higher acceptance of the online version by the respondents.

Lack of standardization and potential for measurement error, notwithstanding, water intake datasets are emerging from various countries worldwide. Iglesia et al. [4], Guelinckx et al. [5], and Ferreira-Pêgo et al. [6] describe absolute and relative water intake in children, adolescents, and adults from surveys with similar 7-day water intake assessment methodologies in 13 countries.

The emerging data signal that low water intake may be an issue of global concern, warranting continued effort to improve assessment techniques, inform, and implement international water intake recommendations. In 12 out of 13 datasets analyzed by Iglesia et al. [4], estimated water intakes are below the EFSA standards for over 20 % of children and adolescents. In many countries, drinking water accounts for less than half of reported fluid intake for children [5]. Overall, only 40 % of men and 60 % of women surveyed appear to meet the acceptable intakes for water intake from fluids as set by EFSA [6].

Guelinckx et al. [7] report that intake volumes of different beverage types differ considerably between countries for adults, but the differences are only modest between countries from the same geographical area. They also observe that intake of free sugar resulting from sweetened beverages exceeds the WHO recommendations on free sugar intake to a different extent between countries. While 11–49 % of adults exceeded this recommendation in Spain, France, Turkey, Iran, Indonesia, and China, 48–62 % of adults were found to exceed the recommendations in Germany, UK, Poland, and Japan, and 60–66 % of adults in Mexico, Brazil, and Argentina.

Accumulating data indicate sub-optimal water intake in multiple countries, and variability in water intake by age, sex, socio-economic status, country, and world region. These findings have potential implications for worldwide obesity and chronic disease risk, and international health disparities. Recent advances in nutrition research on water intake lay the groundwork for further work to understand global beverage intake patterns, trends, determinants, and health consequences.
